# Photon Phase Delay Sensing with Sub-Attosecond Uncertainty

**DOI:** 10.3390/s24072202

**Published:** 2024-03-29

**Authors:** Fabrizio Sgobba, Andrea Andrisani, Luigi Santamaria Amato

**Affiliations:** 1 Italian Space Agency (ASI), Space Geodesy Centre ‘Giuseppe Colombo’, Località Terlecchia, 75100 Matera, MT, Italy; fabrizio.sgobba@asi.it (F.S.); andrea.andrisani@asi.it (A.A.); 2 National Council for Research-National Institute of Optics (CNR-INO), Via Campi Flegrei n. 34, 80078 Pozzuoli, NA, Italy

**Keywords:** Hong–Ou–Mandel interferometry, Cramér–Rao bound, phase delay estimation

## Abstract

The application of statistical estimation theory to Hong–Ou–Mandel interferometry led to enticing results in terms of the detection limit for photon reciprocal delay and polarisation measurement. In the following paper, a fully fibre-coupled setup operating in the telecom wavelength region proves to achieve, for the first time, in common-path Hong–Ou–Mandel-based interferometry, a detection limit for photon phase delay at the zeptosecond scale. The experimental results are then framed in a theoretical model by calculating the Cramer–Rao bound (CRB) and, after comparison with the obtained experimental results, it is shown that our setup attains the optimal measurement, nearly saturating CRB.

## 1. Introduction

Hong–Ou–Mandel (HOM) interference [1] has emerged over the last few decades not only as a fascinating phenomenon in quantum optics but also as a valuable tool within a multitude of applications in the field of quantum metrology, telecommunications, and quantum technology as a whole; see [2] for an accurate review.

When two completely identical (i.e., indistinguishable) photons impinge on the two entry ports of a beam splitter (BS), they interfere, bouncing together and coming out of the same port, as a result of their nature, described by the Bose–Einstein statistics. As a consequence, the coincident events can be seen to drop (ideally to zero) once all distinguishabilities between the photon pair are removed.

The benefit of this phenomenon has become evident across a wide spectrum of applications that range from shaping quantum states [3,4,5] to interferometry and biosensing [6,7,8]. Moreover, the HOM effect has been leveraged efficiently in quantum metrology experiments to generate quantum optical NOON states capable of approaching the Heisenberg limit [9].

A sensor based on the HOM effect presents numerous advantages compared to classical interferometry. The core setup of an HOM interferometer, for one, is notably more robust and simpler to implement since it does not need complex stabilisation mechanisms as the HOM effect is unaffected by the relative phase between the two interfering photons. If classical interferometry is affected by fringe ambiguity whenever applied to measurement ranges greater than half the wavelength of the employed light source due to the periodicity of the acquired signal itself, HOM interferometry allows unambiguous operation over all its interference regions, spanning typically tens or hundreds of wavelengths depending on the spectral profile and frequency of the photons employed (see, for example, [8,10]).

Since HOM interferometry operates at the few-photon level, it is classified as a reliable non-invasive sensing technique, particularly well suited for applications involving biological samples or metrological setups susceptible to damage or thermally induced degradation. In these fields, where light intensity can trigger undesired reactions or compromise the overall accuracy, HOM interferometry results in being the perfect choice, compromising between the high sensitivity and ultra-low optical powers involved.

Over the years, advancements in technology, particularly in single-photon detection [11,12], light engineering [13,14,15], and innovative quantum methodologies [16,17,18,19,20], have continuously improved the performances of HOM-based interferometry [21,22], in turn significantly expanding the scope of its applicability. In 2018, for example, Lyons et al. [23] proposed a quantum-information-based model that allows us to obtain sensitivities as low as ($10^{-18}$ s) in an HOM experiment. In addition to setting a new photon delay measurement record, the aforementioned experiment was performed on photons travelling different paths, de facto overcoming the limitations affecting previous high-resolution experiments, such as [8,10], which were applicable only to photons travelling the same path and hence applicable only to birefringence samples.

Even if often compared to classical interferometry, the HOM effect cannot be seen merely as the low-photon-regime counterpart of classical light interference. This first became evident in studies such as [24,25], which showed how the dip in coincident events (the so-called “photon bouncing”) can still be observed if the photon pair is locally distinguishable when reaching the beam splitter (BS) (hence, where the interference happens) provided that the distinguishability is erased after the BS but before the proper detection [26], akin to the principles of the “spatial” quantum eraser shown in [27,28], which relies on a Young interferometer. Such non-locality, a stranger to classical interferometry, emerges from the quantum nature of the photons involved and can be fully explained only within the framework of quantum optics.

In [29], we applied the idea of [23] to a common-path linear quantum eraser like the ones presented in [8,10], where a resolution of 8 as was obtained (but operating in the telecom range), and a detection limit of 3.8 as was obtained with a partly fibre-coupled setup.

In this work, which can be considered as the natural continuation of [29], using a better-performing apparatus both in terms of state preparation and detection scheme, and inserting the measurement in a theoretical framework not present in [29], we achieved the groundbreaking uncertainty of 187 zs.

## 2. Experimental Setup

**Figure 1:**
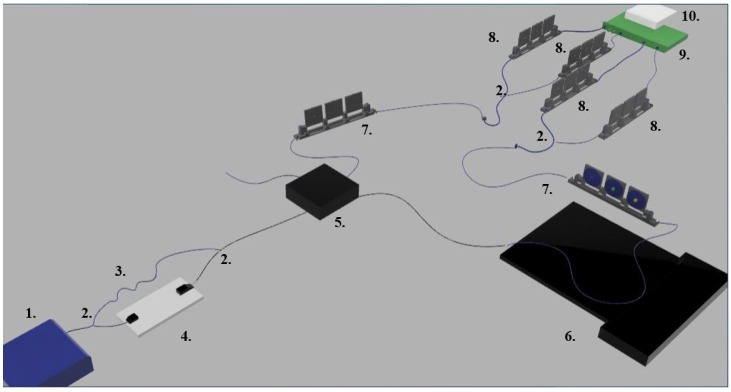
The 3D depiction of the setup employed for temperature-controlled quantum erasing. 1. Twin-photon source; 2. fibre-coupled polarising beam splitter/combiner (PBS); 3. compensatory delay fibre; 4. motorised translation stage with collimated optics; 5. non-polarising fibre-coupled beam splitter (BS); 6. polarisation-maintaining fibre mounted on thermally controlled optical bench; 7. polarisation-rotation paddles for polarisation-information erasure; 8. polarisation-rotation paddles for detector efficiency maximisation; 9. superconductive nanowire single-photon detectors (SNSPDs); and 10. time-tagging electronics.

We realised a fully fibre-coupled temporal quantum eraser that employs a temperature-induced birefringence strain to achieve sub-as detection sensitivities, constituting an absolute landmark for the low-regime quantum metrology literature that revolves around HOM interferometry. The sensor reported in Figure 1 is based on a Twin Photon Source (TPS, 1. in Figure 1), where a Continuous Wave (CW) laser centred at 775 nm is employed to pump a Periodically Poled Lithium–Niobate (PPLN) crystal for Type II Spontaneous Parametric Down Conversion (SPDC) in the telecom region (in particular, the centre emission occurs at 1550 nm). The PPLN crystal is designed to attain its best performance in terms of efficiency and photon indistinguishability at a temperature T=33.9 °C and hence is Peltier-controlled to ensure its long-term stability within these operative conditions.

A symmetrical system constituted by a fibre-coupled polarizing beam splitter and combiner (2. in Figure 1) allows the effective separation of the twin photon pair and acts individually on each photon of the pair in order to restore their time of arrival (ToA) indistinguishability, which is at the core of Hong–Ou–Mandel interference. Hence, one branch of the system acts effectively as a compensatory fixed-delay line, whereas the second branch is equipped with a motorised translation stage that allows the careful tuning of the |H⟩ − |*V*⟩ ToA distinguishability with <0.5 ps-precision on delays.

The heralded photon pair is funneled to one of the inputs of a polarisation-maintaining fibre-coupled (mode *a*) non-polarising beam splitter (BS, 5. in Figure 1) so that the final state encompasses two different longitudinal output modes (c,d).

To retrieve the interference pattern within the HOM dip, one of the two output modes has to be equipped with a tunable polarisation-dependent (i.e., birefringent) delay. In this case, such delay is obtained by purposely heating a 25 cm long polarisation-maintaining fibre, thus gradually modifying its birefringence. The fibre is heated and stabilised in temperature by means of a thermally controlled optical bench (6. in Figure 1).

The erasure of the quantum information stored in the polarisation degree of freedom is performed by equipping polarisation rotation paddles on each output mode. The horizontal and vertical components of light coming out of the erasure paddles are separated and detected using two identical fibre-coupled polarising beam splitters. Each channel is connected to a superconductive nanowire single-photon detector (SNSPD), helium-cooled to a temperature of 2.8 K. Time-tagging electronics (10. in Figure 1) are employed to collect both single-count events and coincident events. Since SNSPD efficiency is strongly dependent on polarisation, each channel has an additional paddle (8. in Figure 1) to maximise light-detector coupling. The setup is mounted on an optical table whose legs are placed on a solid surface under the floors to avoid vibration due to human activity.

## 3. Theoretical Framework

This section will briefly describe the theoretical frame necessary to understand and properly address the linear quantum eraser based on common-path HOM interference, constituting the core of this experiment. Its step-by-step derivation, inspired by works such as [8,10,23,30] but independently retrieved in the exact experimental conditions shown in Figure 1, is reported in the appendices since it falls beyond the proper scope of this paper.

### 3.1. Outcome Probabilities for Delayed-Choice Temporal Quantum Eraser Experiment

Referring to the four detectors as $D_{1}=H_{c}$, $D_{2}=V_{c}$, $D_{3}=H_{d}$ and $D_{4}=V_{d}$, where c,d are the output modes of the BS (5. in Figure 1), the following results are the outcome probabilities of simultaneous detections at different locations (see Appendix A):

(1) $$\begin{array}{c} P\left(D_{1},D_{2}\right)=\frac{1}{8}\left[1-\alpha_{c}\Lambda \left(\frac{2\tau_{1}-2\tau_{0}}{\left|\tau_{-}\right|}\right)\right]\\ P\left(D_{3},D_{4}\right)=\frac{1}{8}\left[1-\alpha_{d}\Lambda \left(\frac{2\tau_{1}-2\tau_{0}+2\tau_{g}}{\left|\tau_{-}\right|}\right)\right]\\ P\left(D_{1},D_{3}\right)=P\left(D_{2},D_{4}\right)=\frac{1}{8}\left[1+\alpha_{cd}\cos(\bar{\omega }\tau )\Lambda \left(\frac{2\tau_{1}-2\tau_{0}+\tau_{g}}{\left|\tau_{-}\right|}\right)\right]\\ P\left(D_{1},D_{4}\right)=P\left(D_{2},D_{3}\right)=\frac{1}{8}\left[1-\alpha_{cd}\cos(\bar{\omega }\tau )\Lambda \left(\frac{2\tau_{1}-2\tau_{0}+\tau_{g}}{\left|\tau_{-}\right|}\right)\right]. \end{array}$$

These will be the only probabilities we will consider in the following, thus disregarding single-count events. In this way, we can avoid loss analysis, as performed in [23], concerning simultaneous detection events misread as single counts due to detector inefficiencies. The function Λ(x) is determined by the convolution between the temporal coherence profile of the pump and the effective length of the nonlinearly active region of the crystal. It therefore presents a triangular shape if the laser pump coherence length is negligible with respect to the crystal length and, conversely, a Lorentzian/Gaussian profile in the opposite case. In general, $\Lambda \left(x\right)$ has its maximum for x=0 where $\Lambda \left(0\right)=1$ (see Appendix A) and tends to zero for x≫1.

With $\tau_{-}$ and $\tau_{0}$ indicated, respectively, the coherence time of the photons’ downconverted emission (hence, $\tau_{-}$ is the FHWH of the function Λ(x)) and the residual birefringent delay are intrinsic in the source itself. $\tau_{1}$, conversely, represents the adjustable polarisation-dependent delay introduced before the beam splitter by means of the motorised translation stage (4. in Figure 1). The quantity $2\bar{\omega }$ is the peak frequency of the optical pump.

The application of a tunable and stabilised temperature across the thermally controlled optical bench (point 6. of Figure 1) and consequently across the polarisation-maintaining fibre (having a temperature dependent birefringence) welded to the bench translates into two polarisation-dependent delays, here reported as $\tau_{g}\left(T\right)$ and τ(T) since they refer to a variation in group or phase velocity of the two orthogonally polarised wavepackets, respectively.

If, therefore, the HOM envelope Λ(x) depends on the ratio of the total time delay due to a group velocity mismatch between photons with different polarisation states, with respect to the global coherence time $\left|\tau_{-}\right|$ of the heralded photon generation inside the crystal, the argument of the cosine function (i.e., the fringe carrier pattern) is given by the relative delay due to the phase velocity mismatch acquired by the photons while crossing the fibre in a given polarisation state with respect to the other one. Finally, $\alpha_{c}$ and $\alpha_{d}$ are the visibility terms for the cases of both photons propagating along the *c* and *d* arms of the beam splitter, respectively (bouncing), while $\alpha_{cd}$ is the visibility term for the case of the photons splitting along two different arms (antibouncing). The visibilities are related to the probability for entangled photons to not experience decoherence when interacting with the experimental apparatus or, more generally, with the environment, and may depend on differences in the spectral profiles, misalignments in the setup, and other intrinsic or experimental issues.

Equation (1) also shows the peculiar characteristic of the delayed-choice temporal quantum eraser. With a postponed delay located at one of the output ports of the beam splitter, one can recover photon indistinguishability by erasing information on the reciprocal delay or on the polarisation even “after” the proper interference happens due to the nonlocal nature of entangled states. Moreover, from such postponed delay arises an interference pattern having the periodicity of the downconverted centre frequency, to which a classical HOM experiment is blind.

Complying with the nature of the setup proposed, in particular with the non-number-resolving detectors, in Equation (1) the probabilities of coincidences within the same detector are discarded. Hence, the ones in Equation (1) do not exhaust all the possible outcomes of the present experiment. In order to treat with normalised probabilities, we conditioned the probabilities in Equation (1) with the event of the simultaneous detection of photons by two different detectors. We then achieve the following new probabilities:

(2) $$p_{ij}\left(\tau \right)=\frac{P\left(D_{i},D_{j}\right)}{\sum_{1\leq k<p\leq 4}P\left(D_{k},D_{p}\right)}=\frac{8\;P\left(D_{i},D_{j}\right)}{6-\alpha_{c}\Lambda \left(\frac{2\tau_{1}-2\tau_{0}}{\left|\tau_{-}\right|}\right)-\alpha_{d}\Lambda \left(\frac{2\tau_{1}-2\tau_{0}+2\tau_{g}}{\left|\tau_{-}\right|}\right)},$$

In (2), we emphasised the fact that the probability distribution $p_{ij}$ depends on the parameter τ, the delay related to the phase velocity birefringence within the heated fibre; such a parameter, if unknown, can be recovered by analyzing the simultaneous detection rates registered during the experiment. All the other parameters appearing in the expressions of $p_{ij}$—those we find in (1)—are supposed to be known and fixed a priori. This is actually not true for the delay relative to the group velocity birefringence $\tau_{g}$ in the fibre. Indeed, the two refractive indexes *n* and $n_{g}$ are related by the equation $n_{g}=n-\lambda dn/d\lambda$, with λ being the photon wavelength.

However, due to the frequency of the downconverted pair $\bar{\omega }\gg 1/\tau_{-}$ (in particular $\bar{\omega }≃1.21\times 10^{15}s^{-1}$ while $\left|\tau_{-}\right|≃4\times 10^{-12}s$), it is possible to identify an operative range for τ of hundreds of fs still restoring a $\tau_{g}/\tau_{-}\ll 1$ and hence an unappreciable shift from the maximum of Λ(x). For the remainder of the paper, therefore, we will assume that Λ does not depend on τ via $\tau_{g}$.

### 3.2. Fisher Information Analysis

Works such as [23,30] proved the efficacy of an information theory approach based on Fisher information analysis to maximise the sensitivity of an HOM-based sensor, obtaining detection limits in the order of magnitude of the as ($10^{-18}$ s), even without interference fringes. The Fisher information function in that case resulted to be $F\left(\tau \right)\propto {\left(1/\tau_{-}\right)}^{2}$, as expected for a sensing apparatus that relies on the HOM envelope. Here, we follow the same successful approach but evaluate the Fisher information for variations in τ within the fringes of the interference pattern, taking full advantage of its intrinsically better sensitivity.

Applying its definition [31], the Fisher information function of the parameter τ for the probability distribution given by (2) reads

(3) $$F\left(\tau \right)=\sum_{1\leq i<j\leq 4}\frac{{\left(dp_{ij}\left(\tau \right)/d\tau \right)}^{2}}{p_{ij}\left(\tau \right)}.$$

and in the hypotheses mentioned in the previous section, after a few calculations we obtain

(4) $$F\left(\tau \right)≃\frac{4{\bar{\omega }}^{2}\alpha_{cd}^{2}\sin^{2}\left(\bar{\omega }\tau \right)\Lambda^{2}\left(\frac{2\tau_{1}-2\tau_{0}+\tau_{g}}{\left|\tau_{-}\right|}\right)}{\left[6-\alpha_{c}\Lambda \left(\frac{2\tau_{1}-2\tau_{0}}{\left|\tau_{-}\right|}\right)-\alpha_{d}\Lambda \left(\frac{2\tau_{1}-2\tau_{0}+2\tau_{g}}{\left|\tau_{-}\right|}\right)\right]\left[1-\alpha_{cd}^{2}\cos^{2}\left(\bar{\omega }\tau \right)\Lambda^{2}\left(\frac{2\tau_{1}-2\tau_{0}+\tau_{g}}{\left|\tau_{-}\right|}\right)\right]}.$$

For $\alpha_{cd}<1$ or $\alpha_{cd}=1$ and Λ<1 Fisher information presents maxima at $\tau_{opt}=\left(\pi /2+k\pi \right)/\bar{\omega }$, with k∈Z, and its maximum has the following expression:

$$F\left(\tau_{opt}\right)≃\frac{4{\bar{\omega }}^{2}\alpha_{cd}^{2}\Lambda^{2}\left(\frac{2\tau_{1}-2\tau_{0}+\tau_{g}}{\left|\tau_{-}\right|}\right)}{6-\alpha_{c}\Lambda \left(\frac{2\tau_{1}-2\tau_{0}}{\left|\tau_{-}\right|}\right)-\alpha_{d}\Lambda \left(\frac{2\tau_{1}-2\tau_{0}+2\tau_{g}}{\left|\tau_{-}\right|}\right)}.$$

**Figure 2:**
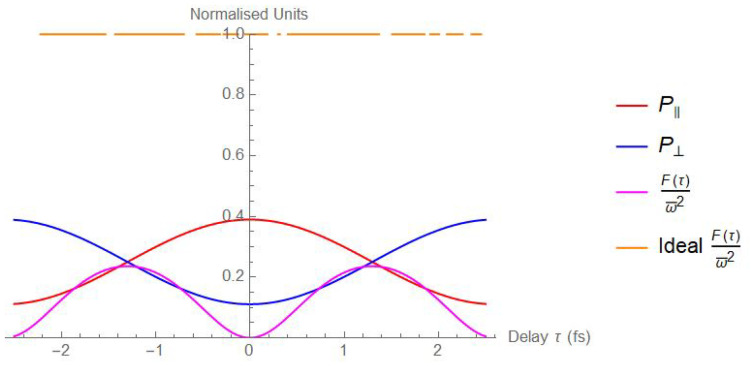
Theoretical evaluation of the functions $P_{\|}$ (red curve), $P_{⊥}$ (blue curve), and $F\left(\tau \right)/{\bar{\omega }}^{2}$ in the experimentally reasonable case $\alpha_{cd}=0.558$, $\alpha_{c}=\alpha_{d}=0.36$ (magenta curve). We added $F\left(\tau \right)/{\bar{\omega }}^{2}$ in the ideal case with $\alpha_{cd}=\alpha_{c}=\alpha_{d}=1$ (orange curve). Everywhere it has been assumed Λ=1.

When $\alpha_{cd}=1$ and Λ=1, on the contrary, *F* is a constant function except where $\tau =\bar{\omega }k\pi$, k∈Z is not defined. So, in perfect experimental conditions, the sensitivity of the final delay sensor is unaffected by the chosen working point. The probabilities $P_{\|}=P\left(D_{1},D_{3}\right)+P\left(D_{2},D_{4}\right)$, $P_{⊥}=P\left(D_{1},D_{4}\right)+P\left(D_{2},D_{3}\right)$, together with the Fisher information function, both with unitary visibilities and with experimentally reasonable visibilities, are reported in Figure 2 in a range τ∈[−2.5fs,2.5fs].

As can be evinced from Figure 2, the non-ideal case F(τ) and the sensitivity of the delay sensor are strongly dependent on the working point chosen.

In order to saturate the Cramér–Rao bound on the variance of τ, it is necessary to find the best estimator $\tau^{*}$ of τ. We used the maximum-likelihood estimator, approaching asymptotically an efficient estimator [32] in the limit of large numbers of samples to find the best resolution point in performing the measurements. The maximum-likelihood estimator (MLE) $\tau^{*}$ is defined implicitly as the solution of the equation

(5) $$\frac{d}{d\tau }\ln L\left(\tau \right)=0,$$

where δt is the acquisition integration time (100 ms) and the likelihood function *L* is defined as $L\left(\tau \right)=\prod_{1\leq i<j\leq 4}p_{ij}{\left(\tau \right)}^{C_{ij}\delta t}$, being $C_{ij},\;1\leq i<j\leq 4$, the experimental coincidence event rates for the detectors $D_{i}$ and $D_{j}$ and *t* the integration time. Solutions of (5) giving a maximum for $L\left(\tau \right)$ result to be

(6) $$\tau^{*}=\pm \frac{1}{\bar{\omega }}\arccos\left[\frac{C_{13}+C_{24}-C_{14}-C_{23}}{\alpha_{cd}\Lambda \left(\frac{2\tau_{1}-2\tau_{0}+\tau_{g}}{\left|\tau_{-}\right|}\right)\left(C_{13}+C_{24}+C_{14}+C_{23}\right)}\right]+\frac{2k\pi }{\bar{\omega }},$$

with k∈Z, hence outlining the typical period ambiguity intrinsic in every fringe-related measurement.

As a consequence of this estimation, it can be demonstrated [32] that the distribution of the MLE $\tau^{*}$ tends for a large number of observations ($N_{obs}$) to a normal distribution around the true parameter value having a variance given by the Cramér–Rao bound (CR)

$$CR_{\tau }\left(N_{obs}\right)=\frac{1}{N_{obs}F\left(\tau \right)}.$$

Such a bound henceforth constitutes the ultimate theoretical limit on the sensitivity achievable on the determination of the parameter ($\sigma_{\tau }^{2}\geq CR_{\tau }$).

## 4. Results and Discussion

### 4.1. Reconstruction of the Hong–Ou–Mandel Feature

In our configuration, we collected events from six different coincidence channels, namely $C_{12}$, $C_{13}$, $C_{14}$, $C_{23}$, $C_{24}$, and $C_{34}$. The following notation can be introduced:

$$C_{s}=C_{12}+C_{34}\;C_{\|}=C_{13}+C_{24}\;C_{⊥}=C_{14}+C_{23}$$

$C_{s}$ refers to events where both photons are transmitted or reflected by the BS, whereas $C_{\|}$ and $C_{⊥}$ refer to events encompassing one transmitted and one reflected photon. The Hong–Ou–Mandel envelope dip/peak can be reconstructed by varying $\tau_{1}$ acting on the motorised translation stage while keeping the temperature on the controlled bench fixed.

**Figure 3:**
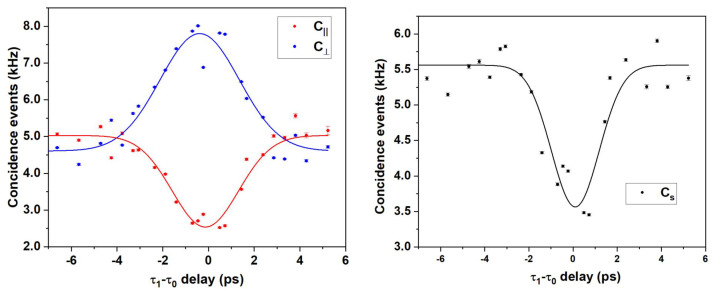
HOM features for $C_{s}$ (black dots), $C_{\|}$ (red dots), and $C_{⊥}$ (blue dots). Each experimental point is the average value of 100 acquisitions. The Gaussian fits (straight lines) are intended to be guides for the eye as well as a tool to determine the visibilities and are not to be intended as a suggestion on the exact shape of Λ(x).

The HOM feature has been reconstructed by acquiring 100 measurements at a 1 s integration time for each single step of the translation stage, spanning a total of 13 ps in 23 steps. The results are plotted in Figure 3, where each dot represents an average over all the 100 acquisitions per step and is reported with its error ($\frac{\sigma }{\sqrt{100}}$), with σ being the standard deviation.

The visibilities retrieved experimentally from the fits in Figure 3 are reported in Table 1 and are expected to be related to the ones reported in Equation (1) by the following equalities:

$$\frac{\alpha_{c}+\alpha_{d}}{2}=\alpha_{s}\;\alpha_{cd}=\alpha_{\|}=\alpha_{⊥}$$

In particular, it can be observed how $\alpha_{⊥}≃\alpha_{\|}$ within 1σ. Hence, the value $\alpha_{cd}$ has been evaluated as the average between $\alpha_{⊥}$ and $\alpha_{\|}$ weighted over their errors ($\sigma_{\|}$, $\sigma_{⊥}$).

$$<\alpha_{cd}{>}_{w}\;=\frac{\alpha_{\|}\cdot \frac{1}{\sigma_{\|}^{2}}+\alpha_{⊥}\cdot \frac{1}{\sigma_{⊥}^{2}}}{\left(\frac{1}{\sigma_{\|}^{2}}+\frac{1}{\sigma_{⊥}^{2}}\right)}=0.558\pm 0.098$$

The distorted shape of the HOM feature, together with low visibilities, could be attributed to non-perfect indistinguishability in the signal and idler spectral profiles. A possible solution may see the implementation of a narrow-band frequency filter. Nonetheless, the resulting temporal broadening, together with a substantial loss of coincident events, would make this solution less appealing for high-sensitivity delay measurements.

### 4.2. Temperature-Induced Interferometric Fringes

In the previous subsection, the HOM envelope profile has been reported, obtained acting on the delay $\tau_{1}$ before the beam splitter. In order to retrieve the interference fringes within the profile, it is necessary to introduce a polarisation-dependent delay in one of its two output modes. Here, such a goal is achieved varying the temperature of a polarisation-maintaining fibre welded to a temperature-controlled optical bench by means of copper scotch in order to ensure the most uniform temperature gradient possible throughout the fibre. For a first measurement, two identical 10 m long polarisation-maintaining fibre spools have been employed. One of the two spools has been welded onto the optical bench, while the other has been kept at room temperature. The fibres were opportunely manufactured so that, once connected, the fast axis of the first one would come to coincide with the slow axis of the second one to compensate the polarisation-dependent delay not related to the asymmetrical temperature variations. The greatest polarisation-dependent delay the two-spool system can provide in this experiment was ∼0.2ps=200fs, set by the maximum temperature gradient allowed for the thermal bench (13.5–42.8 °C).

In agreement with (1), we can see the typical fringe pattern of the coincident events rates, with $C_{\|}$ shifted by half a period with respect to $C_{⊥}$ by varying of the phase delay along the birefringent material placed after the beam splitter. Distance variations between two consecutive fringes are explained by the fact that temperatures in the thermal bench did not change linearly with time. Finally, the fringe amplitude results are almost constant during the acquisition period: according to (1), this confirms our assumption about Λ not varying with τ, at least in the range of τ considered in the experiment.

The two-spool apparatus, which proved useful to witness a 200 fs long quantum-interference pattern (corresponding roughly to 26 wavelengths at 1550 nm), has two major shortcomings when employed for thermal-related delay sensing compared to a shorter polarisation-maintaining fibre: (i) a higher sensitivity to mechanical, vibration, and thermal noise due to its length and its multiple windings and (ii) a which-fringe uncertainty for >0.5 °C temperature variations.

**Figure 4:**
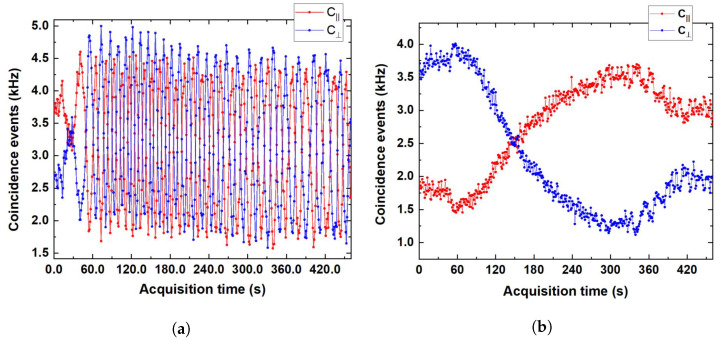
Interference fringes due to temperature-induced birefringence variation in $C_{\|}$ (red dots) and $C_{⊥}$ (blue dots) in the temperature range 13.5–42.8 °C. (**a**) The 10 m long polarisation-maintaining fibre welded on the thermal bench. (**b**) The 25 cm long polarisation-maintaining fibre welded on the thermal bench.

In order to solve these issues, we performed a second experiment in which we welded a short polarisation-maintaining fibre to the thermal bench. The one employed here, 25 cm long, allows us to span a single wavelength in the same temperature interval, as shown in Figure 4b, hence proving more suitable for our purposes.

### 4.3. Calibration of the Delay Sensor

A calibration measurement has been performed repeatedly, stabilising the thermal bench temperature at different values around the $C_{\|/⊥}$ inflection point of the only fringe visible with the 25 cm long polarisation-maintaining fibre welded to the thermal bench, which, as can be observed in Figure 2, corresponds to the maximum of the Fisher information F(τ). For each temperature, we performed 60 acquisitions at a 1 s integration time. The precise assessment of the temperature set for each step has been performed by welding a probe thermometer with copper scotch as close as possible to the heated fibre.

In the previous section, we reported the expression for the best parameter estimator $\tau^{*}$ of the quantity of interest τ (solution of Equation (5)). In the linearity region corresponding to the first fringe, it can therefore be written (Λ≈1)

$$\tau^{*}\approx \frac{1}{\alpha_{cd}\bar{\omega }}\left(\frac{C_{\|}-C_{⊥}}{C_{\|}+C_{⊥}}+\alpha_{cd}\frac{\pi }{2}\right)=\frac{\Delta X}{\alpha_{cd}\bar{\omega }}+\frac{\pi }{2\bar{\omega }}$$

where a new variable has been introduced, ΔX, having its inflection point for ΔX=0.

**Figure 5:**
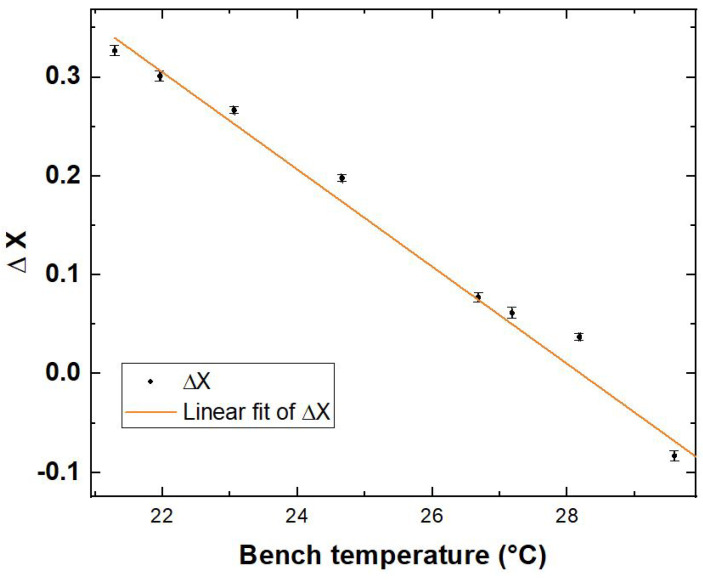
Calibration of the thermally induced delay sensor.

The coincident events for each temperature value have been therefore averaged over the 60 acquisitions, and ΔX has been reported in Figure 5 with its error, obtained propagating the standard deviations on the coincidence events. Data have been fitted with the function (*T* being the temperature measured)

$$\Delta X=aT{(}^{\circ }C)+b$$

Fit parameters *a* and *b* are reported in Table 2 together with the reduced-$\chi^{2}$.

In this region, the best estimation parameter $\tau^{*}$ and the adjustable temperature *T* result in being linearly dependent via the formula

$$\tau^{*}=\frac{a}{\alpha_{cd}\bar{\omega }}T+\frac{b}{\alpha_{cd}\bar{\omega }}+\frac{\pi }{2\bar{\omega }}.$$

Hence, $\tau_{opt}^{*}$ for the first fringe (k=0) results in

$$\tau_{opt}^{*}=\frac{\pi }{2\bar{\omega }}=\frac{a}{\alpha_{cd}\bar{\omega }}T_{opt}+\frac{b}{\alpha_{cd}\bar{\omega }}+\frac{\pi }{2\bar{\omega }}\Rightarrow T_{opt}=-\frac{b}{a}=28.37\;{}^{\circ}C$$

where $T_{opt}$ is the temperature at which can be found the inflection point, which, according to Figure 2, is the working condition that maximises the Fisher information ($\frac{F}{{\bar{\omega }}^{2}}{|}_{T_{opt}}=0.236$), ultimately minimising the detection limit.

### 4.4. Long-Term Measurements

To assess the capabilities of the proposed sensor in terms of temperature-induced delay detection and in order to compare its performance to similar techniques already successfully presented in the recent literature about quantum optics and single-photon optical metrology, we performed a >10 h straight measurement overnight with a 100 ms integration time. The thermal bench temperature was actively stabilised at $T=T_{opt}$. For each measured set of coincidence channels, the variable ΔX has been calculated and successively converted in delays, τ, using the conversion given by the maximum-likelihood estimator $\tau^{*}$, as reported in the previous section. It is a common practice in quantum metrology and sensing [23,30] to perform the long-term stability measurement of the parameter of interest (in this case, the delay, τ) by sequentially hopping between two settings (and consequently in two different parameter values), hence evaluating the differential quantity Δτ, which allows us to efficiently prove the detection limit of the sensor (evaluated as the differential measurement which restores a unitary signal-to-noise ratio) while at the same time removing the common mode mechanical-, thermal-, and source-instability-related drift affecting the parameter measurement in the non-differential configuration.

**Figure 6:**
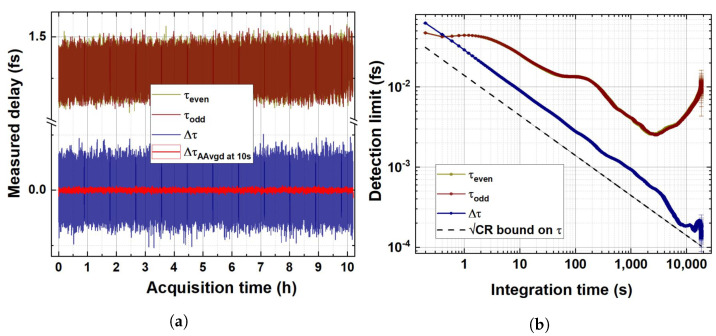
(**a**) Delays in the fixed thermal bench temperature retrieved during a long-term (>10 h) measurement at 100 ms integration time. $\tau_{even}$ (dark yellow) and $\tau_{odd}$ (wine) are superimposed. In navy blue is the differential measurement Δτ. In bright red, the 100-sample adjacent average of Δτ corresponding to an integration time of 10 s is reported. (**b**) Overlapping Allan deviation of the long-term measurement. Color codes between (**a**) and (**b**) are consistent. The Cramér–Rao bound evaluated for a total coincident events rate of 14.6 kHz is also reported in (**b**) as a dashed black line.

In the setup presented in this paper, it is not straightforward to replicate the same procedure since temperature adjustment and stabilisation requires greater dead times than, e.g., current or voltage hopping. Therefore, we sliced our long-term measurement in $\tau_{even}$ and $\tau_{odd}$ values, calculated via even and odd datapoints, respectively, but measured at the same temperature ($T_{opt}$). Hence, our differential measurement should provide a Δτ=0. In Figure 6a, the retrieved delays $\tau_{even}$, $\tau_{odd}$ and Δτ are reported.

In Figure 6b, the overlapping Allan deviation (OAdev) analysis performed on the dataset shown in Figure 6a is reported. The Oadev analysis is compared to the theoretical limit on the detection sensitivity of the variation in the parameter τ, represented by the Cramér–Rao bound (black dotted line), which can be expressed as a function of the integration time *t* as

$$CR_{\tau }{\left(t\right)|}_{\tau =\tau_{opt}}=\frac{1}{F\left(\tau_{opt}\right)N_{obs}}=\frac{1}{F\left(\tau_{opt}\right)\left(<C_{\|}>+<C_{⊥}>+<C_{s}>\right)\cdot t}.$$

The best possible detection limit ($DL_{\tau }$) on single τ is achieved for $t=3\times 10^{3}$ s, resulting in $DL_{\tau }=2.8\times 10^{-3}\;\mathrm{fs}=2.8\;\mathrm{as}$.

As expected, the employment of a differential measurement configuration allows us to cancel oscillations due to the pump instability (integration times 10–500 s) and the long-term thermal drift (integration times $t>2\times 10^{3}$ s) alike, saturating up to 90% of the Cramér–Rao bound CR(t) for $t>7\times 10^{3}$ s and therefore demonstrating experimentally that the estimator $\tau^{*}$ chosen for this paper is the most efficient way possible to employ the proposed detection scheme and leads to the theoretical limit on sensitivity once the aforementioned noise sources are removed. Here, the Δτ detection limit ($DL_{\Delta \tau }$) results in $DL_{\Delta \tau }=0.187\;\mathrm{as}=187\;\mathrm{zs}$ at an integration time of $7.6\times 10^{3}$ s.

The results presented constitute, therefore, to our knowledge, the best results ever achieved in the literature for HOM-based single-photon delay sensing, outperforming the best standards to date [23] and even our previous works [29] by at least an order of magnitude and in a totally fibre-coupled setup meant to be employed in long-scale fibre telecommunication networks.

## 5. Conclusions

In this work, the information theory approach first proposed and implemented by [23] to address the ultimate sensing performance of an HOM-based delay sensor has been extended to a delayed-choice temporal quantum eraser such as the ones proposed by, for example, [8,10]. Additionally, we decided to move our investigations to the telecom region (at 1.55μm), where the fibres show the best transparency window, to address the ever-growing needs of the quantum fibre-telecommunication community. Our setup, totally fibre-coupled and based on a temperature-dependent fibre birifringence to introduce the interference fringe pattern within the HOM dip, is capable of achieving sensitivities at $t>7\times 10^{3}$ of integration time, as low as 187 zs for differential delay measurements, nearly saturating the Cramér–Rao bound and hence qualifying as the best possible experimental procedure to measure postponed birefringent delays with four detectors. The detection limit achieved positions itself as the highest-performing result in the literature of HOM-based quantum metrology, outperforming our previous work [29] and similar results by at least an order of magnitude in a compact setup designed to be “plug and play” from the very start.

## Figures and Tables

**Table 1:** Visibilities of the coincidences $C_{\|}$ ($\alpha_{\|}$), $C_{⊥}$ ($\alpha_{⊥}$), and $C_{s}$ ($\alpha_{s}$). The errors are obtained propagating the fit errors.

$\alpha_{\|}$	$\alpha_{⊥}$	$\alpha_{s}$
0.51±0.07	0.70±0.12	0.36±0.07

**Table 2:** Calibration parameters derived from fit.

*a* (C^−1^)	*b*	Reduced-$\chi^{2}$
−0.049±0.003	1.39±0.08	0.972

## Data Availability

The data that support the findings of this study are available from the corresponding author L.S.A. upon reasonable request.

## Appendix A. Probability Calculations for the Experimental Set-Up of Figure 1

In the present section, we calculate the probabilities for simultaneous detection events with the experimental apparatus described in Figure 1. We will use an approach similar to that adopted in [8]. In the following, we label the photon pair with the Latin letter *i*, with i=1 denoting the vertically polarised photon at the outcomes of the Type II crystal and i=2 for the horizontal one; we indicate the rotation matrix of an angle $\theta_{j}$ along the photon wave vector direction with ${\hat{R}}_{\theta_{j}}$ (j=c,d), describing the actions of the paddles on the polarisation states of the photons passing through arms *c* and *d* of the beam splitter and indicate the vertical and horizontal directions for the two arms of the polarising beam splitters with ${\vec{u}}_{k}$ ($k=1,2,\;{\vec{u}}_{1}=\vec{v},\;{\vec{u}}_{2}=\vec{h}$). Then, the probability density for a pair of photons to cross the paddles on the arms $j_{1}$ and $j_{2}$ of the beam splitter and to propagate along the arms with polarisation ${\vec{u}}_{k_{1}}$ and ${\vec{u}}_{k_{2}}$ will be

(A1) $$p\left(t_{1},\theta_{j_{1}},{\vec{u}}_{k_{1}};t_{2},\theta_{j_{2}},{\vec{u}}_{k_{2}}\right)={\left|\left(\langle t_{1},\theta_{j_{1}},{\vec{u}}_{k_{1}}|\langle t_{2},\theta_{j_{2}},{\vec{u}}_{k_{2}}|\right)|\psi_{I}\rangle \right|}^{2},$$

where $|\psi_{I}\rangle$ describes the quantum state of the entangled photons emerging from the Type II crystal, while $|t_{i},\theta_{j},{\vec{u}}_{k}\rangle$ describe the state of a photon located at time $t_{i}$ along the arm of the polarising beam splitter with orientation ${\vec{u}}_{k}$ after being subjected to a rotation of the polarisation axis of an angle $\theta_{j}$. Regarding $|\psi_{I}\rangle$, we can write

(A2) $$|\psi_{I}\rangle =\;\int \int \int \;\Phi_{\omega_{p}}\left(\omega_{s},\omega_{i}\right)f\left(\omega_{p}\right)\delta \left(\omega_{p}-\omega_{s}-\omega_{i}\right)\left|\vec{h},\omega_{s}\rangle \right|\vec{v},\omega_{i}\rangle \;d\omega_{p}\;d\omega_{s}\;d\omega_{i},$$

where $\omega_{p}$, $\omega_{s}$, and $\omega_{i}$ are, respectively, the the laser pump, the signal, and the idler frequencies; $\Phi_{\omega_{p}}$ is the phase matching function; and $f\left(\omega_{p}\right)$ is the amplitude probability for the laser pump frequency. Reference system orientation is such that the signal and idler photons have horizontal and vertical linear polarisation, respectively. Observe that, by requiring $|\psi_{I}\rangle$ to be normalised, we obtain the following condition for $\Phi_{\omega_{p}}$:

(A3) $$1=\langle \psi_{I}|\psi_{I}\rangle =\;\int \int \;{\left|\Phi_{\omega_{p}}\left(\omega_{s},\omega_{p}-\omega_{s}\right)\right|}^{2}{\left|f\left(\omega_{p}\right)\right|}^{2}d\omega_{s}\;d\omega_{p}.$$

Regarding $|t_{i},\theta_{j},{\vec{u}}_{k}\rangle$ we have

(A4) $$|t_{i},\theta_{j},{\vec{u}}_{k}\rangle ={\hat{E}}_{j}^{\left(-\right)}\left(t_{i},\theta_{j},{\vec{u}}_{k}\right)\left|\mathrm{vac}\rangle =\left[\left({\vec{u}}_{k},{\hat{R}}_{\theta_{j}}\vec{v}\right)\;{\hat{E}}_{j}^{\left(-\right)}\left(t_{i},\vec{v}\right)+\left({\vec{u}}_{k},{\hat{R}}_{\theta_{j}}\vec{h}\right)\;{\hat{E}}_{j}^{\left(-\right)}\left(t_{i},\vec{h}\right)\right]\right|\mathrm{vac}\rangle ,$$

a part of a phase factor. Here, with $\left(\cdot ,\cdot \right)$ we denote the usual scalar product operation, while

(A5) $$\begin{array}{c} {\hat{E}}_{c}^{\left(-\right)}\left(t_{i},\vec{v}\right)=\frac{1}{\sqrt{2\pi }}\int \left\{T^{*}\;e^{i\left[\omega t_{i}-k_{air}\left(\omega \right)L_{\tau_{1}}\right]}{\hat{a}}_{\vec{v}}^{†}\left(\omega \right)+R^{\prime *}\;e^{i\omega t_{i}}{\hat{b}}_{\vec{v}}^{†}\left(\omega \right)\right\}\;d\omega \\ {\hat{E}}_{c}^{\left(-\right)}\left(t_{i},\vec{h}\right)=\frac{1}{\sqrt{2\pi }}\int \left\{T^{*}\;e^{i\omega t_{i}}{\hat{a}}_{\vec{h}}^{†}\left(\omega \right)+R^{\prime *}\;e^{i\omega t_{i}}{\hat{b}}_{\vec{h}}^{†}\left(\omega \right)\right\}\;d\omega \\ {\hat{E}}_{d}^{\left(-\right)}\left(t_{i},\vec{v}\right)=\frac{1}{\sqrt{2\pi }}\int \left\{R^{*}\;e^{i\left[\omega t_{i}-k_{air}\left(\omega \right)L_{\tau_{1}}-k_{f,\vec{v}}\left(\omega \right)L_{f}\right]}{\hat{a}}_{\vec{v}}^{†}\left(\omega \right)+T^{\prime *}\;e^{i\left[\omega t_{i}-k_{f,\vec{v}}\left(\omega \right)L_{f}\right]}{\hat{b}}_{\vec{v}}^{†}\left(\omega \right)\right\}\;d\omega \\ {\hat{E}}_{d}^{\left(-\right)}\left(t_{i},\vec{h}\right)=\frac{1}{\sqrt{2\pi }}\int \left\{R^{*}\;e^{i\left[\omega t_{i}-k_{f,\vec{h}}\left(\omega \right)L_{f}\right]}{\hat{a}}_{\vec{h}}^{†}\left(\omega \right)+T^{\prime *}\;e^{i\left[\omega t_{i}-k_{f,\vec{h}}\left(\omega \right)L_{f}\right]}{\hat{b}}_{\vec{h}}^{†}\left(\omega \right)\right\}\;d\omega \end{array}$$

are creation operators for photons located at time $t_{i}$ along the arms *c* and *d* of the beam splitter in proximity of the paddles. Operators ${\hat{a}}^{†}\left(\omega \right)$ and ${\hat{b}}^{†}\left(\omega \right)$ are creation operators for photons with frequency ω in the a-mode and b-mode, respectively, of the beam splitter, while R,T and $R^{\prime },T^{\prime }$ give the amplitude probabilities for the reflection and transmission of photons in the beam splitter in the a-mode and b-mode. The exponential terms in the above expressions take into account the different paths followed by photons in the experimental apparatus, together with the various media they crossed. $L_{\tau_{1}}$ and $L_{f}$ are, respectively, the (variable) length of the motorised translation stage and the length of the polarisation-maintaining fibre (see Figure 1); $k_{air}\left(\omega \right)$ is the air wave number, while $k_{f,\vec{v}}\left(\omega \right)$ and $k_{f,\vec{h}}\left(\omega \right)$ are the wave number dispersion functions of the fiber for vertically and horizontally polarised photons. Observe that states $|t_{i},\theta_{j},{\vec{u}}_{k}\rangle$ are correctly normalised: indeed, after some simple calculations, we obtain

$$\begin{array}{cc} \; & {\left|\langle t^{\prime },\theta_{c},{\vec{u}}_{k}|t,\theta_{c},{\vec{u}}_{k}\rangle \right|}^{2}=\left({\left|R^{\prime }\right|}^{2}+{\left|T\right|}^{2}\right)\delta \left(t^{\prime }-t\right),\\ \; & {\left|\langle t^{\prime },\theta_{d},{\vec{u}}_{k}|t,\theta_{d},{\vec{u}}_{k}\rangle \right|}^{2}=\left({\left|R\right|}^{2}+{\left|T^{\prime }\right|}^{2}\right)\delta \left(t^{\prime }-t\right), \end{array}$$

and in the case of an ideal beam splitter, we have [33] $\left|T\right|=\left|T^{\prime }\right|$ and

$${\left|R\right|}^{2}+{\left|T\right|}^{2}={\left|R^{\prime }\right|}^{2}+{\left|T^{\prime }\right|}^{2}=1.$$

In the following, since the state $|\psi_{I}\rangle$ does not contain photons in the b-mode, the terms proportional to the creation operator ${\hat{b}}^{†}$ in Equation (A5) will give no contribution to the scalar product (A1); so, from now on, they will be omitted from the expression of ${\hat{E}}^{\left(-\right)}$. Let us now calculate $\left(\langle t_{1},\theta_{c},{\vec{u}}_{k_{1}}|\langle t_{2},\theta_{d},{\vec{u}}_{k_{2}}|\right)|\psi_{I}\rangle$, i.e., the probability density for photons separating at the first beam splitter and propagating along the $u_{k_{1}}$ and $u_{k_{2}}$ arms of the subsequent polarised beam splitters. We have

(A6) $$\begin{array}{cc} \; & \left(\langle t_{1},\theta_{c},{\vec{u}}_{k_{1}}|\langle t_{2},\theta_{d},{\vec{u}}_{k_{2}}|\right)|\psi_{I}\rangle =\langle \mathrm{vac}|{\hat{E}}_{c}^{\left(+\right)}\left(t_{1},\theta_{c},{\vec{u}}_{k_{1}}\right){\hat{E}}_{d}^{\left(+\right)}\left(t_{2},\theta_{d},{\vec{u}}_{k_{2}}\right)\psi_{I}\rangle =\\ \; & =\frac{RT}{2\pi }\langle \mathrm{vac}|\;\int \int \;\;\;d\omega_{1}d\omega_{2}\left\{\left({\vec{u}}_{k_{1}},{\hat{R}}_{\theta_{c}}\vec{v}\right)\;e^{-i\left[\omega_{1}t_{1}-\phi_{\vec{v},1}\left(\omega_{1}\right)\right]}{\hat{a}}_{\vec{v}}\left(\omega_{1}\right)+\left({\vec{u}}_{k_{1}},{\hat{R}}_{\theta_{c}}\vec{h}\right)\;e^{-i\omega_{1}t_{1}}{\hat{a}}_{\vec{h}}\left(\omega_{1}\right)\right\}\cdot \\ \; & \;\left\{\left({\vec{u}}_{k_{2}},{\hat{R}}_{\theta_{d}}\vec{v}\right)\;e^{-i\left[\omega_{2}t_{2}-\phi_{\vec{v},2}\left(\omega_{2}\right)\right]}{\hat{a}}_{\vec{v}}\left(\omega_{2}\right)+\left({\vec{u}}_{k_{2}},{\hat{R}}_{\theta_{d}}\vec{h}\right)\;e^{-i\left[\omega_{2}t_{2}-\phi_{\vec{h},2}\left(\omega_{2}\right)\right]}{\hat{a}}_{\vec{h}}\left(\omega_{2}\right)\right\}\psi_{I}\rangle , \end{array}$$

where ${\hat{E}}_{j}^{\left(+\right)}$ are the adjoint operators of ${\hat{E}}_{j}^{\left(-\right)}$ and

(A7) $$\begin{array}{c} \phi_{\vec{v},1}\left(\omega_{1}\right)=k_{air}\left(\omega_{1}\right)L_{\tau_{1}}\\ \phi_{\vec{v},2}\left(\omega_{2}\right)=k_{air}\left(\omega_{2}\right)L_{\tau_{1}}+k_{f,\vec{v}}\left(\omega_{2}\right)L_{f}\\ \phi_{\vec{h},2}\left(\omega_{2}\right)=k_{f,\vec{h}}\left(\omega_{2}\right)L_{f}. \end{array}$$

From (A2), the double application of the annihilation operators ${\hat{a}}_{\vec{v}}$ and ${\hat{a}}_{\vec{h}}$ to $|\psi_{i}\rangle$, required in (A6), gives the following results:

$$\begin{array}{c} {\hat{a}}_{\vec{v}}\left(\omega_{1}\right){\hat{a}}_{\vec{v}}\left(\omega_{2}\right)|\psi_{I}\rangle =0\\ {\hat{a}}_{\vec{h}}\left(\omega_{1}\right){\hat{a}}_{\vec{h}}\left(\omega_{2}\right)|\psi_{I}\rangle =0\\ {\hat{a}}_{\vec{v}}\left(\omega_{1}\right){\hat{a}}_{\vec{h}}\left(\omega_{2}\right)\left|\psi_{I}\rangle =\;\int \int \;\Phi_{\omega_{p}}\left(\omega_{s},\omega_{p}-\omega_{s}\right)f\left(\omega_{p}\right)\delta \left(\omega_{1}-\omega_{p}+\omega_{s}\right)\delta \left(\omega_{2}-\omega_{s}\right)\;d\omega_{p}\;d\omega_{s}\right|\mathrm{vac}\rangle \\ {\hat{a}}_{\vec{h}}\left(\omega_{1}\right){\hat{a}}_{\vec{v}}\left(\omega_{2}\right)\left|\psi_{I}\rangle =\;\int \int \;\Phi_{\omega_{p}}\left(\omega_{s},\omega_{p}-\omega_{s}\right)f\left(\omega_{p}\right)\delta \left(\omega_{1}-\omega_{s}\right)\delta \left(\omega_{2}-\omega_{p}+\omega_{s}\right)\;d\omega_{p}\;d\omega_{s}\right|\mathrm{vac}\rangle , \end{array}$$

so that (A6) becomes

(A8) $$\begin{array}{cc} \; & \left(\langle t_{1},\theta_{c},{\vec{u}}_{k_{1}}|\langle t_{2},\theta_{d},{\vec{u}}_{k_{2}}|\right)|\psi_{I}\rangle =\frac{RT}{2\pi }\int \;\;\int d\omega_{p}\;d\omega_{s}e^{-i\omega_{p}t_{1}}\Phi_{\omega_{p}}\left(\omega_{s},\omega_{p}-\omega_{s}\right)f\left(\omega_{p}\right)\cdot \\ \; & \left\{\left({\vec{u}}_{k_{1}},{\hat{R}}_{\theta_{c}}\vec{v}\right)\left({\vec{u}}_{k_{2}},{\hat{R}}_{\theta_{d}}\vec{h}\right)e^{-i\left[\omega_{s}\left(t_{2}-t_{1}\right)-\phi_{\vec{v},1}\left(\omega_{p}-\omega_{s}\right)-\phi_{\vec{h},2}\left(\omega_{s}\right)\right]}+\right.\\ \; & \;\left.+\left({\vec{u}}_{k_{1}},{\hat{R}}_{\theta_{c}}\vec{h}\right)\left({\vec{u}}_{k_{2}},{\hat{R}}_{\theta_{d}}\vec{v}\right)e^{-i\left[\left(\omega_{p}-\omega_{s}\right)\left(t_{2}-t_{1}\right)-\phi_{\vec{v},2}\left(\omega_{p}-\omega_{s}\right)\right]}\right\}\;. \end{array}$$

By inserting (A8) in (A1), we can determine the probability density $p\left(t_{1},\theta_{c},{\vec{u}}_{k_{1}};t_{2},\theta_{d},{\vec{u}}_{k_{2}}\right)$ and then the probability

(A9) $$P\left(\theta_{c},{\vec{u}}_{k_{1}};\theta_{d},{\vec{u}}_{k_{2}}\right)=\;\int \int \;p\left(t_{1},\theta_{c},{\vec{u}}_{k_{1}};t_{2},\theta_{d},{\vec{u}}_{k_{2}}\right)\;dt_{1}\;dt_{2}$$

where the integrals are calculated over all possible photon arrival times $t_{1},\;t_{2}$ within the coincidence resolving time. Usually, the resolving time set in a coincidence counting experimental apparatus is much greater then the mutual coherence time of the two-photon wave packet. So, we can extend, without adding significant sources of errors, the time integrals in (A9) from −∞ to +∞. In this case, taking into account that

$$\left({\vec{u}}_{k_{1}},{\hat{R}}_{\theta_{c}}\vec{v}\right)\left({\vec{u}}_{k_{2}},{\hat{R}}_{\theta_{d}}\vec{h}\right)\left({\vec{u}}_{k_{1}},{\hat{R}}_{\theta_{c}}\vec{h}\right)\left({\vec{u}}_{k_{2}},{\hat{R}}_{\theta_{d}}\vec{v}\right)=l\left({\vec{u}}_{k_{1}},{\vec{u}}_{k_{2}}\right)\frac{\sin2\theta_{c}\sin2\theta_{d}}{2},$$

where

$$l\left({\vec{u}}_{k_{1}},{\vec{u}}_{k_{2}}\right)=\left\{\begin{array}{ccc} 1 & \mathrm{if} & {\vec{u}}_{k_{1}}\|{\vec{u}}_{k_{2}}\\ -1 & \mathrm{if} & {\vec{u}}_{k_{1}}⊥{\vec{u}}_{k_{2}}. \end{array}\right.$$

After a few calculations (A9) becomes

(A10) Pθc,u→k1;θd,u→k2=RT2u→k1,R^θcv→2u→k2,R^θdh→2+u→k1,R^θch→2u→k2,R^θdv→2++lu→k1,u→k2sin2θcsin2θd2Re∫dωpfωp2∫dωsΦωpωs,ωp−ωsΦωp*ωp−ωs,ωs·expiϕv→,1ωp−ωs−ϕv→,2ωs+ϕh→,2ωs.

The first term on the right of (A10) gives the probability of an event in which the photon with vertical polarisation propagates along the *c* arm of the beam splitter, while the photon with horizontal polarisation propagates along the *d* arm; the opposite is given by the second term. Finally, the third one is the interference term between these two alternatives. This purely quantum term arises due to the entanglement between the two photons emerging from the crystal; nevertheless, some couples of photon entanglement can be lost due to decoherence effects caused by the interaction of the photons with the experimental apparatus or more generally with the ambiance. In this case, if $1-\alpha_{cd}$ is the probability for a couple of photons to arrive in a non-entangled state at the detectors—actually, at the polarising beam splitter—by taking into account decoherence effects, we can re-write (A10) as

(A11) Pθc,u→k1;θd,u→k2==RT221+luk1,uk2−cos2θccos2θd+αcdsin2θcsin2θdRe∫dωpfωp2·∫−∞+∞dΩΓωpΩe−iϕv→,2ωp2+Ω−ϕv→,1ωp2−Ω−ϕh→,2ωp2+Ω,

where we also performed the change of variable $\omega_{s}\to \omega_{p}/2+\Omega$, and we defined a new function

(A12) $$\Gamma_{\omega_{p}}\left(\Omega \right)=\Phi_{\omega_{p}}\left(\frac{\omega_{p}}{2}+\Omega ,\frac{\omega_{p}}{2}-\Omega \right)\Phi_{\omega_{p}}^{*}\left(\frac{\omega_{p}}{2}-\Omega ,\frac{\omega_{p}}{2}+\Omega \right),$$

and used the fact that

$${\left({\vec{u}}_{k_{1}},{\hat{R}}_{\theta_{c}}\vec{v}\right)}^{2}{\left({\vec{u}}_{k_{2}},{\hat{R}}_{\theta_{d}}\vec{h}\right)}^{2}+{\left({\vec{u}}_{k_{1}},{\hat{R}}_{\theta_{c}}\vec{h}\right)}^{2}{\left({\vec{u}}_{k_{2}},{\hat{R}}_{\theta_{d}}\vec{v}\right)}^{2}=\frac{1-l\left({\vec{u}}_{k_{1}},{\vec{u}}_{k_{2}}\right)\cos2\theta_{1}\cos2\theta_{2}}{2}.$$

The parameter $\alpha_{cd}$ is the said visibility term. Let us now assume that the exponent functions inside (A11) are slowly varying with respect to Ω; so, in the case $\Gamma_{\omega_{p}}\left(\Omega \right)$ is almost null outside a small neighbourhood centred in zero, we can develop such functions around $\omega_{p}/2$ in Taylor series arrested at the first order. From Equations (A7), after a few calculations, we obtain

(A13) $$\begin{array}{cc} \; & \phi_{\vec{v},2}\left(\frac{\omega_{p}}{2}+\Omega \right)-\phi_{\vec{v},1}\left(\frac{\omega_{p}}{2}-\Omega \right)-\phi_{\vec{h},2}\left(\frac{\omega_{p}}{2}+\Omega \right)=\\ \; & \;=2\Omega \frac{L_{\tau_{1}}}{c}+\left[k_{f,\vec{v}}\left(\frac{\omega_{p}}{2}\right)-k_{f,\vec{h}}\left(\frac{\omega_{p}}{2}\right)\right]L_{f}+\Omega \left[\frac{dk_{f,\vec{v}}}{d\omega }{|}_{\frac{\omega_{p}}{2}}-\frac{dk_{f,\vec{h}}}{d\omega }{|}_{\frac{\omega_{p}}{2}}\right]L_{f}=\\ \; & \;=\Omega \left(2\tau_{1}+\tau_{g}\right)+\frac{\omega_{p}}{2}\tau , \end{array}$$

where

$$\begin{array}{ccc} \tau_{1} & = & \frac{L_{\tau_{1}}}{c}\\ \tau_{g} & = & \left[\frac{dk_{f,\vec{v}}}{d\omega }{|}_{\frac{\omega_{p}}{2}}-\frac{dk_{f,\vec{h}}}{d\omega }{|}_{\frac{\omega_{p}}{2}}\right]L_{f}\\ \tau & = & \frac{2}{\omega_{p}}\left[k_{f,\vec{v}}\left(\frac{\omega_{p}}{2}\right)-k_{f,\vec{h}}\left(\frac{\omega_{p}}{2}\right)\right]L_{f}=\left[n_{f,\vec{v}}\left(\frac{\omega_{p}}{2}\right)-n_{f,\vec{h}}\left(\frac{\omega_{p}}{2}\right)\right]\frac{L_{f}}{c}. \end{array}$$

In the following, we will modify the first term on the right of (A13) by adding $-2\tau_{0}$ inside the square brackets. This term will take into account the intrinsic birefringence coming from the source. By inserting (A13) in (A11), we obtain

(A14) $$\begin{array}{cc} \; & P\left(\theta_{c},{\vec{u}}_{k_{1}};\theta_{d},{\vec{u}}_{k_{2}}\right)=\\ \; & \frac{{\left|RT\right|}^{2}}{2}\left[1+l\left({\vec{u}}_{k_{1}},{\vec{u}}_{k_{2}}\right)\left(-\cos2\theta_{1}\cos2\theta_{2}+\alpha_{cd}K\left(\tau ,2\tau_{1}-2\tau_{0}+\tau_{2}\right)\sin2\theta_{1}\sin2\theta_{2}\right)\right], \end{array}$$

where

(A15) Kτ,2τ1−2τ0+τ2=2πRe∫−∞∞dωpfωp2Γ˜ωp2τ1−2τ0+τ2e−iωpτ2==2π∫−∞∞dωpfωp2Γ˜ωp2τ1−2τ0+τ2cosωpτ2,

with ${\tilde{\Gamma }}_{\omega_{p}}$ the Fourier transform of $\Gamma_{\omega_{p}}$. The last equality in (A15) is due to the fact that, by (A12), $\Gamma_{\omega_{p}}{\left(\Omega \right)}^{*}=\Gamma_{\omega_{p}}\left(-\Omega \right)$, so that the function ${\tilde{\Gamma }}_{\omega_{p}}$ is real. If the function $f\left(\omega_{p}\right)$ is extremely peaked around $2\bar{\omega }$, that is, ${\left|f\left(\omega_{p}\right)\right|}^{2}≃\delta \left(\omega_{p}-2\bar{\omega }\right)$, we have

$$K\left(\tau ,2\tau_{1}-2\tau_{0}+\tau_{2}\right)=\sqrt{2\pi }\;{\tilde{\Gamma }}_{2\bar{\omega }}\left(2\tau_{1}-2\tau_{0}+\tau_{2}\right)\cos\left(\bar{\omega }\tau \right).$$

Regarding the function ${\tilde{\Gamma }}_{2\bar{\omega }}$, if the crystal length is much greater than the laser pump coherence length, for the phase matching function $\Phi_{\omega_{p}}$ of (A15), we have

(A16) $$\begin{array}{cc} \; & \Phi_{\omega_{p}}\left(\bar{\omega }+\Omega ,\bar{\omega }-\Omega \right)=C\;\mathrm{sinc}\left(\left[k_{e}\left(\bar{\omega }+\Omega \right)+k_{o}\left(\bar{\omega }-\Omega \right)-k_{p}\left(2\bar{\omega }\right)\right]\frac{L}{2}\right)≃\\ \; & \;≃C\;\mathrm{sinc}\left(\frac{\tau_{-}\Omega }{2}\right). \end{array}$$

Here, *C* is a normalisation constant, *L* is the crystal length, $k_{e}$, $k_{o}$, and $k_{p}$ are, respectively, the signal, idler, and pump beam wave numbers inside the Type II crystal,

$$\tau_{-}=\left[\frac{dk_{e}}{d\omega }{|}_{\bar{\omega }}-\frac{dk_{o}}{d\omega }{|}_{\bar{\omega }}\right]L,$$

and the last equality in (A16) follows from momentum conservation equation $k_{e}\left(\bar{\omega }\right)+k_{o}\left(\bar{\omega }\right)=k_{p}\left(2\bar{\omega }\right)$, after expanding $k_{e}$ and $k_{o}$ around $\bar{\omega }$ in Taylor series up to the first order. By applying (A3), we obtain $C=\sqrt{\frac{\left|\tau_{-}\right|}{2\pi }}$. Then, after few calculations, we achieve

$$\begin{array}{cc} \; & {\tilde{\Gamma }}_{2\bar{\omega }}\left(2\tau_{1}-2\tau_{0}+\tau_{2}\right)=\frac{\left|\tau_{-}\right|}{{\left(2\pi \right)}^{3/2}}\int_{-\infty }^{+\infty }\mathrm{sinc}{\left(\frac{\tau_{-}\Omega }{2}\right)}^{2}e^{-i\Omega \left(2\tau_{1}-2\tau_{0}+\tau_{2}\right)}\;d\Omega =\\ \; & \;=\frac{1}{\sqrt{2\pi }}\Lambda \left(\frac{2\tau_{1}-2\tau_{0}+\tau_{2}}{\left|\tau_{-}\right|}\right), \end{array}$$

where

$$\Lambda \left(x\right)=\left\{\begin{array}{ccc} 1-\left|x\right| & \mathrm{if} & \left|x\right|\leq 1\\ 0 & \mathrm{if} & \left|x\right|>1 \end{array}\right.$$

is a triangular function. So, for *K* we have

(A17) $$K\left(\tau ,2\tau_{1}-2\tau_{0}+\tau_{2}\right)=\Lambda \left(\frac{2\tau_{1}-2\tau_{0}+\tau_{2}}{\left|\tau_{-}\right|}\right)\cos\left(\bar{\omega }\tau \right).$$

In the following, we will adopt (A17) even when laser pump coherence length and crystal length are generic; in this case, the Λ function will not be a triangular function but a more generic one—for example, a Lorentz or Gaussian function—, with $\left|\tau_{-}\right|$ the scale factor determining the appreciable variations in Λ at the varying of its argument $2\tau_{1}-2\tau_{0}+\tau_{g}$. Additionally, since Λ is the Fourier transform of the square modulus of a normalised function, we have $\Lambda \left(0\right)=1$. With all these assumption, probability $P\left(\theta_{c},{\vec{u}}_{k_{1}};\theta_{d},{\vec{u}}_{k_{2}}\right)$ finally reads

(A18) $$\begin{array}{cc} \; & P\left(\theta_{c},{\vec{u}}_{k_{1}};\theta_{d},{\vec{u}}_{k_{2}}\right)=\\ \; & \frac{{\left|RT\right|}^{2}}{2}\left[1+l\left({\vec{u}}_{k_{1}},{\vec{u}}_{k_{2}}\right)\left(-\cos2\theta_{c}\cos2\theta_{d}+\alpha_{cd}\Lambda \left(\frac{2\tau_{1}-2\tau_{0}+\tau_{2}}{\left|\tau_{-}\right|}\right)\cos\left(\bar{\omega }\tau \right)\sin2\theta_{c}\sin2\theta_{d}\right)\right]. \end{array}$$

In the same manner, we can determine the probabilities for photon pairs to propagate along the same arm of the beam splitter. Indeed, it can be shown that at the *c* arm of the beam splitter, that is, the transmission one with respect to the input arm *a*, we achieve the following detection probabilities:

(A19) $$P\left(\theta_{c};{\vec{u}}_{k_{1}},{\vec{u}}_{k_{2}}\right)=\frac{{\left|T\right|}^{4}}{4}\left[m\left({\vec{u}}_{k_{1}},{\vec{u}}_{k_{2}}\right)+l\left({\vec{u}}_{k_{1}},{\vec{u}}_{k_{2}}\right)\left(1+\alpha_{c}\Lambda \left(\frac{2\tau_{1}-2\tau_{0}}{\left|\tau_{-}\right|}\right)\right)\sin^{2}2\theta_{d}\right],$$

where we considered a different visibility term $\alpha_{c}$ and

$$m\left({\vec{u}}_{k_{1}},{\vec{u}}_{k_{2}}\right)=\left\{\begin{array}{ccc} 0 & \mathrm{if} & {\vec{u}}_{k_{1}}\|{\vec{u}}_{k_{2}}\\ 4 & \mathrm{if} & {\vec{u}}_{k_{1}}⊥{\vec{u}}_{k_{2}} \end{array}\right.,\;l^{\prime }\left({\vec{u}}_{k_{1}},{\vec{u}}_{k_{2}}\right)=\left\{\begin{array}{ccc} l\left({\vec{u}}_{k_{1}},{\vec{u}}_{k_{2}}\right) & \mathrm{if} & {\vec{u}}_{k_{1}}\|{\vec{u}}_{k_{2}}\\ 2l\left({\vec{u}}_{k_{1}},{\vec{u}}_{k_{2}}\right) & \mathrm{if} & {\vec{u}}_{k_{1}}⊥{\vec{u}}_{k_{2}}, \end{array}\right.$$

For the *d* arm of the beam splitter—the reflection one with respect to the *a* arm—we achieve

(A20) $$P\left(\theta_{d};{\vec{u}}_{k_{1}},{\vec{u}}_{k_{2}}\right)=\frac{{\left|R\right|}^{4}}{4}\left[m\left({\vec{u}}_{k_{1}},{\vec{u}}_{k_{2}}\right)+l\left({\vec{u}}_{k_{1}},{\vec{u}}_{k_{2}}\right)\left(1+\alpha_{d}\Lambda \left(\frac{2\tau_{1}+2\tau_{2}-2\tau_{0}}{\left|\tau_{-}\right|}\right)\right)\sin^{2}2\theta_{d}\right].$$

By summing all the probabilities reported in (A18), we achieve the overall probability for the pair of the heralded photons coming from the Type II crystal to separate at the first beam splitter:

(A21) $$P\left(\theta_{c},\theta_{d}\right)=P\left(\theta_{c},\vec{v};\theta_{d},\vec{v}\right)+P\left(\theta_{c},\vec{v};\theta_{d},\vec{h}\right)+P\left(\theta_{c},\vec{h};\theta_{d},\vec{v}\right)+P\left(\theta_{c},\vec{h};\theta_{d},\vec{h}\right)=2{\left|R\right|}^{2}{\left|T\right|}^{2}$$

By repeating the same sum for the probabilities reported in (A19) and (A20), we obtain the bouncing probabilities at the *c* and *d* arm of the beam splitter

(A22) $$P\left(\theta_{c}\right)=P\left(\theta_{c};\vec{v},\vec{v}\right)+P\left(\theta_{c};\vec{v},\vec{h}\right)+P\left(\theta_{c};\vec{h},\vec{h}\right)={\left|T\right|}^{4}$$

(A23) $$P\left(\theta_{d}\right)=P\left(\theta_{d};\vec{v},\vec{v}\right)+P\left(\theta_{d};\vec{v},\vec{h}\right)+P\left(\theta_{d};\vec{h},\vec{h}\right)={\left|R\right|}^{4}.$$

Observe that $P\left(\theta_{c};\vec{h},\vec{v}\right)$ and $P\left(\theta_{d};\vec{h},\vec{v}\right)$ refer to the same events of $P\left(\theta_{c};\vec{v},\vec{h}\right)$ and $P\left(\theta_{d};\vec{v},\vec{h}\right)$, respectively, so they were omitted in (A22) and (A23). By summing (A21)–(A23), we obtain

$$P\left(\theta_{c},\theta_{d}\right)+P\left(\theta_{c}\right)+P\left(\theta_{d}\right)={\left({\left|R\right|}^{2}+{\left|T\right|}^{2}\right)}^{2},$$

with ${\left({\left|R\right|}^{2}+{\left|T\right|}^{2}\right)}^{2}$ equal to 1 in the case of an ideal beam splitter. Finally, by setting

$${\left|T\right|}^{2}={\left|R\right|}^{2}=1/2,\;\theta_{c}=\theta_{d}=\pi /4$$

in (A18)–(A20), we achieve the probabilities reported in Section 3.

## References

[1] Hong C.K., Ou Z.Y., Mandel L. (1987). Measurement of subpicosecond time intervals between two photons by interference. Phys. Rev. Lett..

[2] Bouchard F., Sit A., Zhang Y., Fickler R., Miatto F.M., Yao Y., Sciarrino F., Karimi E. (2020). Two-photon interference: The Hong–Ou–Mandel effect. Rep. Prog. Phys..

[3] Ulanov A.E., Fedorov I.A., Sychev D., Grangier P., Lvovsky A.I. (2016). Loss-tolerant state engineering for quantum-enhanced metrology via the reverse Hong–Ou–Mandel effect. Nat. Commun..

[4] Chen Y., Ecker S., Wengerowsky S., Bulla L., Joshi S.K., Steinlechner F., Ursin R. (2018). Polarization Entanglement by Time-Reversed Hong-Ou-Mandel Interference. Phys. Rev. Lett..

[5] Chen Y., Ecker S., Chen L., Steinlechner F., Huber M., Ursin R. (2021). Temporal distinguishability in Hong-Ou-Mandel interference for harnessing high-dimensional frequency entanglement. npj Quantum Inf..

[6] Ndagano B., Defienne H., Branford D., Shah Y.D., Lyons A., Westerberg N., Gauger E.M., Faccio D. (2022). Quantum microscopy based on Hong–Ou–Mandel interference. Nat. Photon..

[7] Dorfman K.E., Asban S., Gu B., Mukamel S. (2021). Hong-Ou-Mandel interferometry and spectroscopy using entangled photons. Commun. Phys..

[8] Branning D., Migdall A.L., Sergienko A.V. (2000). Simultaneous measurement of group and phase delay between two photons. Phys. Rev. A.

[9] Dowling J.P. (2008). Quantum optical metrology—The lowdown on high-N00N states. Contemp. Phys..

[10] Dauler E., Jaeger G., Muller A., Migdall A., Sergienko A. (1999). Tests of a two-photon technique for measuring polarization mode dispersion with subfemtosecond precision. J. Res. Natl. Inst. Stand. Technol..

[11] Russo S.D., Elefante A., Dequal D., Pallotti D.K., Amato L.S., Sgobba F., de Cumis M.S. (2022). Advances in Mid-Infrared Single-Photon Detection. Photonics.

[12] Nomerotski A., Keach M., Stankus P., Svihra P., Vintskevich S. (2020). Counting of Hong-Ou-Mandel Bunched Optical Photons Using a Fast Pixel Camera. Sensors.

[13] Walborn S.P., de Oliveira A.N., Pádua S., Monken C.H. (2003). Multimode Hong-Ou-Mandel Interference. Phys. Rev. Lett..

[14] D’Ambrosio V., Carvacho G., Agresti I., Marrucci L., Sciarrino F. (2019). Tunable Two-Photon Quantum Interference of Structured Light. Phys. Rev. Lett..

[15] Kim H., Lee S.M., Kwon O., Moon H.S. (2017). Two-photon interference of polarization-entangled photons in a Franson interferometer. Sci. Rep..

[16] Yepiz-Graciano P., Martínez A.M.A., Lopez-Mago D., Cruz-Ramirez H., U’Ren A.B. (2020). Spectrally resolved Hong–Ou–Mandel interferometry for quantum-optical coherence tomography. Photonics Res..

[17] Triggiani D., Psaroudis G., Tamma V. (2023). Ultimate Quantum Sensitivity in the Estimation of the Delay between two Interfering Photons through Frequency-Resolving Sampling. Phys. Rev. Appl..

[18] Jin R.B., Gerrits T., Fujiwara M., Wakabayashi R., Yamashita T., Miki S., Terai H., Shimizu R., Takeoka M., Sasaki M. (2015). Spectrally resolved Hong-Ou-Mandel interference between independent photon sources. Opt. Express.

[19] Kobayashi T., Ikuta R., Yasui S., Miki S., Yamashita T., Terai H., Yamamoto T., Koashi M., Imoto N. (2016). Frequency-domain Hong–Ou–Mandel interference. Nat. Photonics.

[20] Orre V.V., Goldschmidt E.A., Deshpande A., Gorshkov A.V., Tamma V., Hafezi M., Mittal S. (2019). Interference of Temporally Distinguishable Photons Using Frequency-Resolved Detection. Phys. Rev. Lett..

[21] Xue Y., Yoshizawa A., Tsuchida H. (2010). Hong-Ou-Mandel dip measurements of polarization-entangled photon pairs at 1550 nm. Opt. Express.

[22] Tsujimoto Y., Wakui K., Fujiwara M., Sasaki M., Takeoka M. (2021). Ultra-fast Hong-Ou-Mandel interferometry via temporal filtering. Opt. Express.

[23] Lyons A., Knee G.C., Bolduc E., Roger T., Leach J., Gauger E.M., Faccio D. (2018). Attosecond-resolution Hong-Ou-Mandel interferometry. Sci. Adv..

[24] Pittman T.B., Strekalov D.V., Migdall A., Rubin M.H., Sergienko A.V., Shih Y.H. (1996). Can Two-Photon Interference be Considered the Interference of Two Photons?. Phys. Rev. Lett..

[25] Kwiat P.G., Steinberg A.M., Chiao R.Y. (1992). Observation of a “quantum eraser”: A revival of coherence in a two-photon interference experiment. Phys. Rev. A.

[26] Wheeler J.A. (1978). The “Past” and the “Delayed-Choice” Double-Slit Experiment. Mathematical Foundations of Quantum Theory.

[27] Scully M.O., Drühl K. (1982). Quantum eraser: A proposed photon correlation experiment concerning observation and “delayed choice” in quantum mechanics. Phys. Rev. A.

[28] Kim Y.H., Yu R., Kulik S.P., Shih Y., Scully M.O. (2000). Delayed “Choice” Quantum Eraser. Phys. Rev. Lett..

[29] Sgobba F., Andrisani A., Dello Russo S., Siciliani de Cumis M., Santamaria Amato L. (2023). Attosecond-Level Delay Sensing via Temporal Quantum Erasing. Sensors.

[30] Harnchaiwat N., Zhu F., Westerberg N., Gauger E., Leach J. (2020). Tracking the polarisation state of light via Hong-Ou-Mandel interferometry. Opt. Express.

[31] Kay S.M. (1993). Statistical Signal Processing: Estimation Theory.

[32] Wolfowitz J. (1965). Asymptotic efficiency of the maximum likelihood estimator. Theory Probab. Its Appl..

[33] Hamilton M.W. (2000). Phase shifts in multilayer dielectric beam splittersp. Am. J. Phys..

